# Kinetic Aspects of Esterification and Transesterification in Microstructured Reactors

**DOI:** 10.3390/molecules29153651

**Published:** 2024-08-01

**Authors:** Xingjun Yao, Zhenxue Wang, Ming Qian, Qiulin Deng, Peiyong Sun

**Affiliations:** 1 Shandong Provincial Key Laboratory of Chemical Energy Storage and Novel Cell Technology, School of Chemistry and Chemical Engineering, Liaocheng University, Liaocheng 252059, China; 2 School of Materials Science and Engineering, Southwest University of Science and Technology, Mianyang 621010, China; qiulindeng@swust.edu.cn; 3 Beijing Institute of Petrochemical Technology, Daxing District, Beijing 102617, China; sunpeiyong@bipt.edu.cn

**Keywords:** kinetic determination, microreactors, microfluidic, esterification, transesterification

## Abstract

Microstructured reactors offer fast chemical engineering transfer and precise microfluidic control, enabling the determination of reactions’ kinetic parameters. This review examines recent advancements in measuring microreaction kinetics. It explores kinetic modeling, reaction mechanisms, and intrinsic kinetic equations pertaining to two types of microreaction: esterification and transesterification reactions involving acids, bases, or biocatalysts. The utilization of a micro packed-bed reactor successfully achieves a harmonious combination of the micro-dispersion state and the reaction kinetic characteristics. Additionally, this review presents micro-process simulation software and explores the advanced integration of microreactors with spectroscopic analyses for reaction monitoring and data acquisition. Furthermore, it elaborates on the control principles of the micro platform. The superiority of online measurement, automation, and the digitalization of the microreaction process for kinetic measurements is highlighted, showcasing the vast prospects of artificial intelligence applications.

## 1. Background

Reaction kinetics play a critical role in optimizing synthetic processes. They hinge on the interaction between reaction and mixing rates, which relies on conditions and mixing efficiency. To secure reliable kinetic data, it is crucial to maintain high mixing efficiency, precise temperature control, and a narrow residence time distribution (RTD).

Kinetic studies, similar to parameter screening, aim to model system behavior under optimized conditions, primarily developing a mathematical correlation between the reaction rate and significant parameters, including pressure, temperature, and concentration. This involves determining the reaction order, estimating rate-constant parameters like pre-exponential factor (A) and activation energy (Ea), and distinguishing between different rate models using data from carefully designed experiments. Kinetic modeling becomes more nuanced when sequentially selecting experiments with an appropriate algorithm in order to maximize reaction information. The literature presents two predominant types of kinetic expressions: empirical and mechanistically based. The former is derived from regression analyses of well-designed experiments but is specific to certain conditions, while the latter leverages the understanding of chemical mechanisms to formulate elementary rate expressions. Assumptions regarding the rate-determining step often facilitate a kinetic expression that captures a significant portion of data variability. This kinetic information is vital when scaling up processes [1].

Empirical rate laws, derived from the regression analysis of thorough experiments, are confined to the specific conditions under which they were derived. Alternatively, the use of chemical mechanisms to derive elementary rate expressions is another method. Through strategic assumptions about the rate-determining step, a kinetic expression encapsulating a substantial portion of data variability can often be established.

The crux of chemical research lies in unraveling the mechanisms of chemical reactions. Even for seemingly straightforward reactions, the intermediate steps remain initially unknown, as intermediates are typically short-lived and elude direct observation. Comprehensive knowledge of a reaction’s kinetic mechanism aids in designing superior chemical systems, enabling the identification of advantageous reactant characteristics and facilitating the development of better reagents and catalysts that are engineered to lower the transition state energy required for critical bond breaking. Such an understanding also leads to more precise and robust kinetic expressions [2].

Kinetic analysis of multistep organic reactions is crucial for understanding its mechanisms and practically applying organic synthesis. These studies determine concentration dependencies, rate constants, and equilibrium constants for elementary steps, striving to fully elucidate reaction pathways. Nonetheless, experimental data analysis in multistep catalytic reactions poses challenges due to the complexity of specific rate laws, leading researchers to often seek simplified approaches with which to represent kinetic data [3].

Acquiring precise reaction rate parameters in batch laboratory equipment often presents challenges. Industries such as pharmaceuticals and fine chemicals, restricted by limited reactant availability, struggle to accumulate sufficient data for reaction modeling due to deficient information and flawed experimental designs. Integrating in situ analytical techniques such as spectroscopy and calorimetry into batch reactors can enrich the information obtained about each experiment. However, the uneven distribution of concentrations and temperatures in these systems may cause deviation, particularly in volatile or reflux reactions, where the liquid solution and headspace concentrations are uncertain. Traditional laboratory-scale reactors, with pressure-resistant thick walls, suffer from heat transfer resistances, which obscures the true reaction kinetics by impairing catalyst performance. Microreactors, possessing higher heat-mass transfer coefficients and specific surface areas mitigates these issues, as confirmed by the dimensionless parameters of Hatta number (Ha) and Thiele modulus (Φ). These microsystems deliver rapid heat and mass transfer, generating improved reaction profiles and facilitating more precise kinetic studies than traditional batch processes [4].

Continuous-flow chemistry techniques have shown substantial waste reduction, stimulating interest in bioconversion processes where recent developments are enhancing biocatalytic processes. Integrating continuous-flow platforms with in-line analytics yields significant benefits, merging flow chemistry efficiency with swift data acquisition concerning chemical, structural, and process aspects [5]. Automated microreactors, with real-time data collection capabilities, facilitate unsupervised sequential experiments and the self-optimization of yield or E-factor. The Model-Based Design of Experiments (MBDoE) aids in efficient experiment design, improving model comprehension regarding optimal parameter estimation. This system allows researchers to delve into reaction mechanisms in granular detail, providing insights into the progression of trans (esterification) reactions and facilitating reaction optimization and deployment [6].

Overall, the combination of flow microreactors, in-line analytics, and advanced modeling techniques enhances the understanding of reaction kinetics, facilitates reaction optimization, and offers new avenues for sustainable and efficient chemical synthesis. Here, we report the advancements made in the kinetic determination aspect of esterification and transesterification in microreactors over the decade. This paper is organized into the following sections: 1—Background; 2—The calculation of kinetic parameters; 3—The kinetic of esterification and transesterification in microstructured reactors; 4—Software simulation; 5—Microreactions online; 6—Automation and digitization reactions; 7—Conclusions.

## 2. Calculation of Kinetic Parameters

### 2.1. Kinetic Parameters Determination

Kinetic data generation involves two methods: sampling steady-state conditions in a flow setting or generating time-series data in a batch. The analysis of experimental data in reaction kinetics primarily employs integration and differentiation methods. Through these approaches, we can establish connections between the reaction rate and parameters like concentrations, temperature, and pressure. Various papers [7,8] extensively elucidate the reaction rate constant and its dependencies. These resources serve as valuable references for comprehending the core principles of reaction kinetics and offer guidance for the precise and practical compelling analysis of experimental data.

$$r=\frac{dC_{A}}{dt}=f(C_{A},C_{B},C_{C},\ldots T,P)={\mathrm{kC}}_{A}^{\alpha }C_{B}^{\beta }C_{C}^{\gamma }\ldots \;k=A\exp(-E_{a}/RT)$$

### 2.2. Flow Manipulation Method for Kinetic Parameters Determination in a Microfluidic System

**Figure 1:**
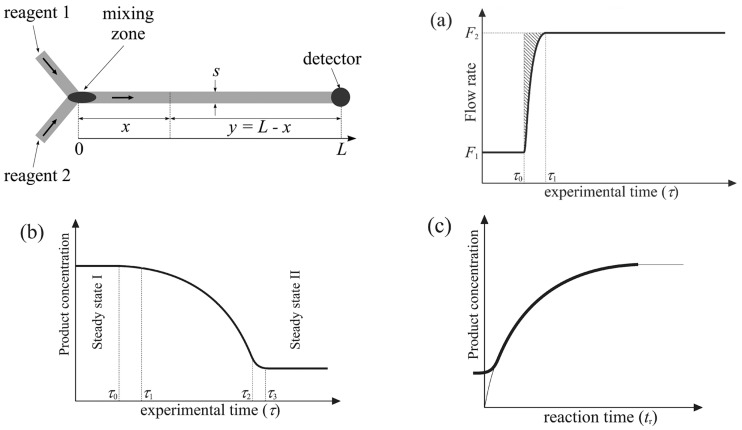
Changes in (**a**) flow rate and (**b**) the detected product concentration occurring during the proposed procedure and (**c**) the calculated kinetic curve. L is the length of the flow path, x is any distance along the flow path, s is the channel cross-section, and total volume V = sL. y is the distance from any position x to the measurement point at time τ_0_ [18].

To achieve multiple kinetic results in a single test, microfluidic technology employs the non-steady-state operation mode. This approach involves a step of increasing flow rate F_1_ to F_2_, coupled with real-time detection that is conducted at the outlet of the flow tube. This setup enables the acquisition of location-specific information without physically moving the detection spot along the microreactor channel (Figure 1).

To determine the kinetics, the flow rate and temperature in the material conveying system and temperature control system are continuously varied with time, following a pre-set program known as the flow rate ramping method (Equation (1)). As the liquid flow rate declines, the residence time progressively increases. Throughout this process, the inline detector captures the continuous changes in signals, allowing for the determination of reactant conversion at different residence times [9,10,11,12,13,14,15,16,17].

(1) $$\begin{array}{l} t_{r}=-\frac{F_{2}-F_{1}}{F_{1}}\tau +\frac{F_{2}}{F_{1}}\tau_{2},\tau_{1}=\tau_{3}-\frac{V}{F_{2}},F_{eff}=(1+\alpha_{V}\Delta T)F\\ \frac{1}{{\left[Bz\right]}_{tr}^{n-1}}-\frac{1}{{\left[Bz\right]}_{0}^{n-1}}=(n-1)kt_{r},\frac{1}{{\left[Bz\right]}^{n-1}}-\frac{1}{{\left[Bz\right]}_{0}^{n-1}}=-\frac{\left(n-1)k(F_{2}-F_{1}\right)}{F_{1}}\tau +\frac{(n-1)kF_{2}}{F_{1}}\tau_{2} \end{array}$$

Residence time, tr; the effective flow rate in the microreactor, F_eff_; the volumetric coefficient of expansion, α_V_. [Bz]_tr_ and [Bz]_0_ are the reactant concentration in the mixture at time tr and the initial time, respectively [18].

Similarly, temperature variations can effectively determine Ea in a single experiment. The integration of transient temperature ramping and inline analysis facilitates the investigation of reaction selectivity and kinetics. Obviously, varying temperature ranges unveil diverse rate-determining steps, leading to disparate selectivity outcomes. Importantly, during the residence time range, temperature fluctuations should be disregarded, assuming a homogeneous temperature field for the reaction. Thus, the temperature ramping rate should be appropriately synchronized with the residence time to ensure accurate results.

The flow rate ramping method boasts several benefits: it enables kinetic equation and parameter derivation from a single test, lowers reagent use, and reduces the experiment’s duration. Depending on the flow rate ratio (F_2_/F_1_), it provides spatial information; shortens measurement times, particularly for slow reactions; and bypasses photochemical effects and localized heating, which tied to optical methods.

### 2.3. Michaelis–Menten Equation and the Kinetic Parameters

The Michaelis–Menten, Lineweaver–Burk, and Ping Pong Bi Bi (PPBB) kinetic models describe the kinetic properties of enzyme-catalyzed reactions and the interactions between enzymes and substrates [19]. They help us to understand enzyme catalytic mechanisms, evaluate drug potency, and design and optimize bio-packed-bed reactors and enzymatic processes.

#### 2.3.1. Michaelis–Menten, Lineweaver–Burk Kinetic Models

The Michaelis–Menten plot is a widely used model for describing enzymatic reaction rates [20]. At low substrate concentrations, the reaction follows first-order kinetics. In the intermediate range, the reaction exhibits mixed-order kinetics concerning the substrate. However, as the substrate concentration increases, the reaction shifts from first-order to zero-order kinetics. The Michaelis–Menten plot is an essential tool for understanding the behavior of enzymatic reactions and their dependence on substrate concentration.

The enzyme kinetics obey the Michaelis–Menten equation for describing their behavior:

$$V_{0}=\frac{V_{\max}[S]}{\left(K_{m}+[S]\right)}$$

The Lineweaver–Burk equation and the Lilly–Hornby model are described in Table 1.

#### 2.3.2. Ping Pong Bi Bi Mechanism Kinetic Model

In the PPBB mechanism, two substrates, denoted as A and B, bind to the enzyme sequentially, followed by the release of two products, denoted as P and Q. This involves two distinct kinetic steps: firstly, substrate A binds to the enzyme, forming an enzyme–substrate complex (EA). This is followed by the subsequent binding of substrate B to the complex, forming a ternary complex (EAB). The reaction between the substrates then takes place within the ternary complex, forming of products P and Q. Finally, the products are released from the enzyme, regenerating the free enzyme for use in further catalytic cycles. The PPBB mechanism is described in Table 1.

## 3. Kinetic of Esterification and Transesterification in Microrstructured-Reactors

ZF Yan et al. [8,19,20,25,26,27] report various kinetic determination methods and fundamental patterns of various reaction types. These findings have significant applications in organic synthesis, pharmaceutics, proteomics, and biotechnology. In the following sections, we focus on the determination method of microreaction kinetics, explicitly focusing on esterification and transesterification, examining their operational aspects and theoretical mechanisms.

### 3.1. Kinetic of Esterification

**Figure 2:**
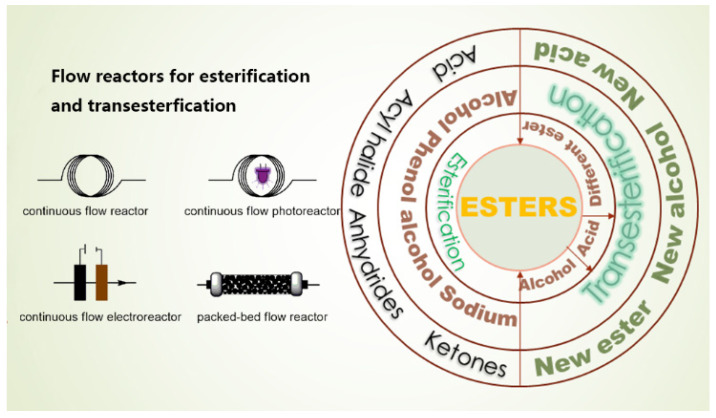
Flow reactors (**Left**) and relationship (**Right**) with esterification and transesterification.

In addition to the direct esterification reaction between acids and alcohols, esters can also be formed through other types of reactions: acyl halides reacting with alcohols, phenols, and sodium alcohols; anhydrides reacting with alcohols, phenols, and sodium alcohols; and ketones reacting with alcohols, phenols, and sodium alcohols, etc. (Figure 2). The following image describes the different types of esterification kinetic.

#### 3.1.1. Liquid Acid or Base-Catalyzed Esterification

In a spiral microreactor, the synthesis of Butyl Levulinate (BL) was performed, achieving a maximum conversion of 89.19% and a BL selectivity of 92% under the following conditions: 35% (mole) sulphuric acid catalyst loading, 2 min of space time, and a fixed mole ratio of 1:8 (LA:BOH) at 60 °C. The microreactor’s efficacy was appraised via RTD studies utilizing a pulse tracer input. Analyzing C and E curves denoted a negative skewness of 48.25 s^2^, suggesting minimal axial dispersion and a lack of dead zones. Equivalence between the system’s mean residence and space times pointed towards nearly ideal functioning. The dispersion number, standing at 0.0062, affirmed the system’s adherence to a plug flow regime. Assuming plug flow behavior, the kinetic velocity constant was determined (K = 0.002004 M^−1^s^−1^) and fitted to a second-order pseudo-homogeneous model [28]. This is shown in Equation (2) below.

(2) $$\begin{array}{l} \mathrm{LA}+\mathrm{BOH}\overset{H^{+}}{⇄}{\mathrm{BL}+H}_{2}O\;(W)\\ \tau =\frac{V}{v_{0}}=\frac{VC_{LA0}}{F_{LA0}}=C_{LA0}\int_{0}^{X_{LA}}\frac{dX_{LA}}{-r_{A}},\begin{array}{c} \;-r_{A}=KC_{LA}C_{BOH} \end{array},\begin{array}{c} \;C_{BOH}= \end{array}C_{LA0}(M-X_{LA}),\\ -r_{A}=KC_{LA0}^{2}(1-X_{LA})(M-X_{LA}),\begin{array}{c} \;M=\frac{C_{BOHO}}{C_{LA0}} \end{array},\begin{array}{c} \;Ln(\frac{M-X_{LA}}{M(1-X_{LA})} \end{array})=KC_{LA0}(M-1)\tau \end{array}$$

The esterification kinetics equation of LA and BOH.

**Figure 3:**
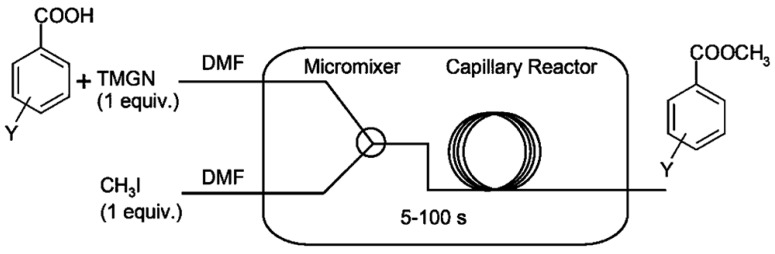
Experimental setup. It comprises a commercially available micromixer (nanomixer) and a fused silica-based capillary tubular reactor [29].

Gholamipour-Shirazi A et.al conducted benzoic acid alkylation in a microfluidic reactor, using iodomethane in N, N′-dimethylformamide (DMF) with the superbase 1,8-bis-(tetramethyl guanidino) naphthalene (TMGN) as a deprotonation agent (Figure 3) [29]. The feeding method of the employed second-order kinetics reaction was as follows: we added TMGN + MeI and benzoic acid feed separately. The proton sponge base used, TMGN, exhibited sluggish alkylation under our specific conditions, and the combination of feeding demonstrated a high sensitivity to residual water. Over a six-magnitude range in rate-constant fluctuations, the conversion-flow rate correlation demonstrated linearity across all temperatures, signifying the prompt attainment of thermal equilibrium. In benzoic acid alkylation with MeI in DMF, the reactivity displayed a strong correlation with the Hammett reaction constant (−0.65). Using less than 0.5 mmol of substrate per condition, the reaction rates were measured. The Arrhenius diagram and Hammett free-energy relationships’ robust correlations confirmed the efficacy of capillary continuous-flow microreactors. Utilizing the rate constants obtained, an Arrhenius plot was constructed, which displayed linearity (R^2^ = 0.993) within the temperature range of 4–70 °C. By investigating the temperature dependence and linear free-energy relationships of the alkylation reaction, valuable insights into its mechanism can be obtained, including the determination of E_a_ (43.1 kJ·mol^−1^), enthalpies, and entropies. This information plays a crucial role in identifying the rate-limiting step and discerning the intermediates involved in the reaction. Moreover, establishing a Hammett linear relationship for a series of substituted benzoic acids shows that the bulky halogens are more reactive and that alkyl substituents are less reactive, thereby potentially suggesting modifications that can optimize its efficiency.

#### 3.1.2. Wall-Coated Catalyst for Esterification

Amin Delparish et al. used a Au/SiO_2_-coated microchannel reactor to the execute oxidative esterification of furfural in methanol, leveraging molecular O_2_ across a vast operational ambit, including explosive regions. They examined the reaction rate’s reliance on variables such as O_2_ partial pressure, furfural concentration, temperature, and the amount of additive base used, with an eye on potential mass transfer effects. Utilizing the collected kinetic data and ab initio density functional theory calculations, they discerned and detailed the pivotal reaction stages. The principal role of O_2_ was hypothesized as enabling the revitalization of active sites via hydrogen abstraction from the gold surface. Furthermore, the breakdown of methanol into methoxy species on Au sites was considered to be a significant step in the reaction sequence, notably affecting the overall reaction rate. In contrast, furfural’s involvement in the rate-determining step was negated. Methanol is dehydrogenated to the methoxy group on gold. This is considered to be an important determinant, affecting the overall reaction rate and reducing the demand for additional alkali [30]. Some other studies on esterification kinetics are listed in Table 2.

#### 3.1.3. Biocatalytic Esterification

Biocatalytic processes are widely recognized as highly cost-efficient and sustainable methods for producing valuable biological compounds. These processes often involve devices that incorporate enzymes that are immobilized on beads or microfluidic channel walls, although some utilize dissolved enzymes within the microfluidic system to drive reactions. While specific model systems predominantly employ enzymes such as glucose oxidase, horseradish peroxidase, and alkaline phosphatase, microreactors find significant application in fields like the tryptic digestion of proteins and polymerase chain reactions for the automated analysis of proteomic and genetic materials, respectively. Moreover, enzymatic microreactors serve as valuable tools for characterizing enzyme activity based on substrate concentration, and for facilitating the rapid screening of novel biocatalysts and their substrates. Furthermore, they play pivotal roles in lab-on-a-chip techniques and ATAS, contributing to biomolecule analysis [48,49,50].

Microreactors’ exceptional efficiency in enzymatic reactions is attributed to their laminar fluid flow in microchannels, enabling accurate process control, quick mixing, and brief residence time. The benefits of laminar flow include improved heat and mass transfer rates and a significant surface-to-volume ratio, reducing enzyme consumption during optimization. This culminates in accelerated reaction rates, enhanced yields, and productivity, effectively driving down costs. The high degree of parallelization achievable through microfluidics allows for the rapid testing of diverse process conditions within a short timeframe [39]. Moreover, microreactors are instrumental in assessing enzyme-catalyzed reactions’ kinetic parameters by eliminating radial diffusion constraints, allowing for the accurate estimation of intrinsic kinetics. Sokač Cvetni’c et al. scrutinized the integration of enzymatic processes’ reaction kinetics and mathematical modeling in microreactors, shedding light on characteristics like reduced area and laminar flow [19].

Microreactors play a crucial role in the online monitoring and kinetic characterization of biocatalytic processes, particularly for supported enzymes. When immobilized, enzymes exhibit improved stability compared to that of their free-solution counterparts. Microreactors offer a unique advantage by enabling the determination of K_m_ and V_max_ for these immobilized enzymes. Miniaturized systems operating in continuous-flow mode are beneficial in characterizing newly immobilized enzymes. These systems require minimal immobilized enzymes and can be easily automated, making them highly efficient. Significantly, such methods overcome the challenges associated with batch assays for immobilized enzymes, such as difficulties in correctly mixing the solid particles and substrate solutions containing the loaded enzyme [26].

**Figure 4:**
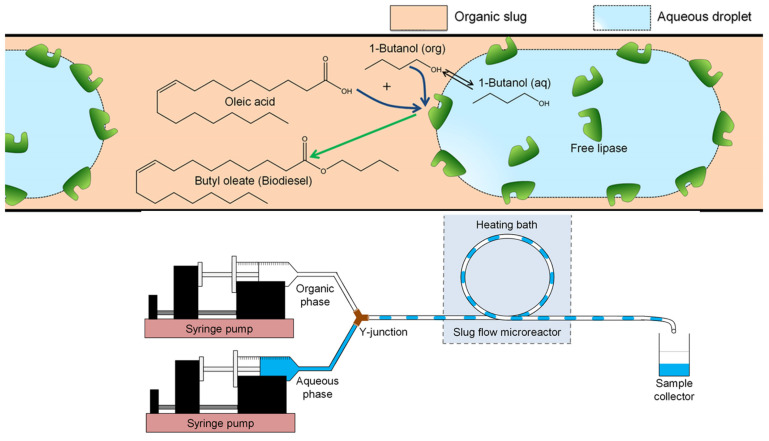
Graphical overview of free lipase-catalyzed oleic acid esterification in slug flow microre actor (with hydro phobic wall). The enzyme was dissolved in the aqueous phase, oleic acid in the organic (n-heptane) phase, and 1-butanol distributed over the two phases with the reaction takingplace on the aqueous−organic interface. Schematic presentation of PTFE microreactor setup [24].

When exploring oleic acid and 1-butanol enzymatic esterification in capillary microreactors, aqueous and organic phases were channeled into a polyether ether ketone (PEEK) Y-junction with a 0.5 mm inner diameter, creating an aqueous–organic slug flow in the ensuing polytetrafluoroethylene (PTFE) microreactor (Figure 4). Here, the Rhizomucormiehei lipase in the aqueous phase acted as the catalyst, with n-heptane serviced as the organic solvent. Within 30 min at 30 °C, almost-complete conversion into butyloleate occurred. The heightened reaction rate in smaller microreactors could be ascribed to the larger interfacial area, facilitating increased enzyme activation at the interface. The reaction kinetics within the PTFE microreactor, influenced by kinetic variables such as enzyme and substrate concentration, interfacial area, and the flow ratio of aqueous-to-organic volume, aligned with the kinetic model with a PPBB mechanism during competitive 1-butanol inhibition. The hydrophilic stainless-steel microreactor demonstrated a superior enzyme turnover at higher organic volume fractions than the hydrophobic PTFE counterpart, underscoring its potential for process intensification [24].

**Figure 5:**
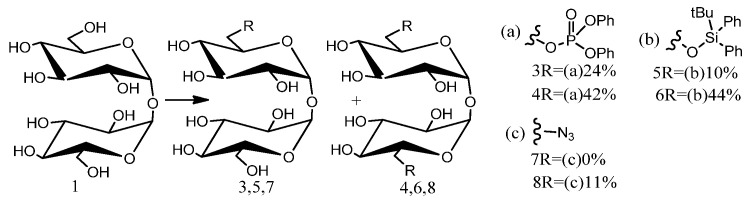
Conditions: (**a**) diphenyl chlorophosphate, py, 18 h; (**b**) tert-butyldiphenyl chlorosilane, imidazole, DMF, 26 h; (**c**) HN_3_, PPh_3_, diisopropyl-azodicarboxylate, 1,4-dioxane, 66 h.

Mitul K. Patel et al.’s application of flow chemistry techniques to investigate trehalose desymmetrization produced critical kinetic data, elucidating the mechanisms of these ostensibly simple reactions. Techniques for trehalose dissolution improvement include reaction mixture dilution and secondary hydroxyl group protection (Figure 5). Alternatively, the reverse modification method utilizes phase effects, yielding beyond-statistical mono-functionalization expectations. This approach, combined with the facile and scalable synthesis of compound **5**, improves the overall yield and facilitates its inclusion in non-symmetrical synthetic routes made of trehalose analog. Its potential applications extend to pseudosymmetric disaccharide polyols, enabling selective primary hydroxyl group modifications. Under high (low) solubility, k_1_ and k_2_ were determined to be 21.9 × 10^−3^ and 17.9 × 10^−3^ mol^−1^dm^3^s^−1^ (21.6 × 10^−3^, 7.67 × 10^−3^ mol^−1^dm^3^s^−1^), respectively (Equation (3)) [51].

(3) $$\begin{array}{l} \mathrm{Tre}+R\overset{k_{1}}{\to }Mono+R\overset{k_{2}}{\to }Di\\ d[Tre]/dt=-k_{1}[Tre][R],d[Mono]/dt=-k_{1}[Tre][R]-k_{2}[Mono][R]\\ d[Di]/dt=k_{2}[Mono][R],d[R]=-k_{1}[Tre][R]-k_{2}[Mono][R] \end{array}$$

Flow chemistry kinetic of the unsymmetrical trehalose analogs.

**Figure 6:**
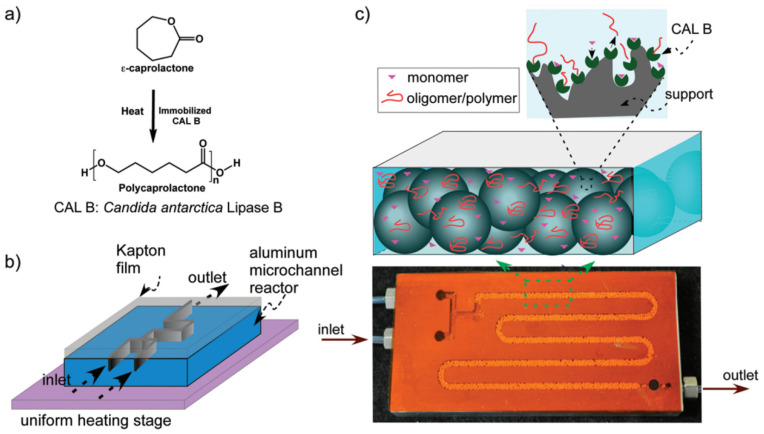
(**a**) Ring-opening polymerization reaction of ε-caprolactone. (**b**) Schematic of aluminum microreactor covered with Kapton film using thermally cured epoxy. Microreactor was placed on uniform heating stage for temperature control. (**c**) Image shows CALB-immobilized solid beads were filled in channel. Polymerization reactions took place at enzyme active sites [52].

Antanu Kundu et al.’s study applied microreactors to the explore enzyme-catalyzed ring-opening polymerization of ε-caprolactone into polycaprolactone. Implementing an innovative microreactor design (Figure 6), heterogeneous reactions were executed continuously within organic media at high temperatures. This approach facilitated faster polymerization rates and higher molecular masses than batch reactors. Apparent rate constants ranged from 0.007 to 0.012 s^−1^, indicating first-order kinetics [52].

#### 3.1.4. Solid Acid- or Base-Catalyzed Esterification in Packed-Bed Reactors (PBRs)

Solid acid or base-catalyzed esterification predominantly occurs in a micro-PBR. Essentially, a PBR or fixed-bed reactor (FBR) is a cylindrical tube packed with catalyst pellets, facilitating the conversion of reactants into products as they flow through. The catalyst may feature diverse configurations, such as a single large bed, multiple horizontal beds, or parallel packed tubes. The primary function of a packed bed is to enhance inter-phase contact in chemical processes, rendering it useful in chemical reactors.

**Figure 7:**
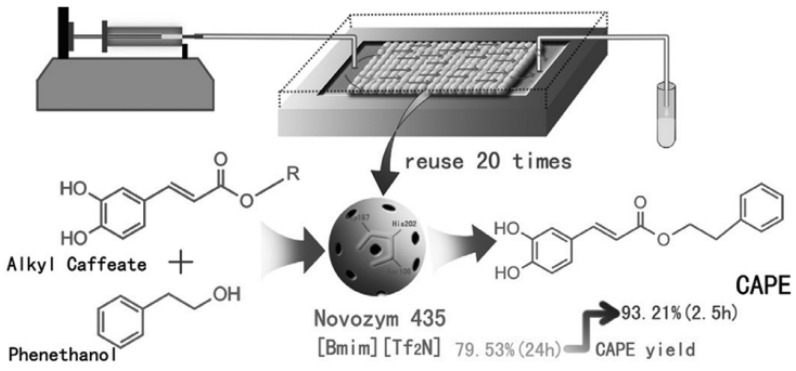
Continuous-flow biosynthesis of caffeic acid phenethyl ester in PBR [53].

In the industrial production of a caffeic acid phenethyl ester (CAPE), a PBR (Figure 7) is employed for the Novozym 435 catalyzed esterification of methyl caffeate acid (CA) and 2-phenylethanol (PE) in [Bmim] [Tf_2_N] ionic liquids. Remarkably, using a PBR resulted in a CAPE yield of 93.21% within 2.5 h, whereas a batch reactor required 24 h to achieve the exact yield. The K_m(app)_ values obtained for the microreactors ranged from 14.04 to 39.56 mM. Notably, the Novozym 435 enzyme maintained its activity, even after 20 cycles of reuse and continuous operation for nine days [53].

Riky Lim et al. investigated biodiesel production from R. trisperma oil, particularly the esterification step optimization using Lewatit K2640 as a catalyst while maintaining a constant methanol concentration. The study found that temperature, catalyst loading, and the methanol-to-oil molar ratio individually significantly impacted the acid value of the esterified oil, with catalyst loading being the most influential factor. Conversely, the interaction between these factors did not significantly affect the acid value. R. trisperma kinetics were examined based on the assumption of pseudo-homogeneous second-order kinetics, yielding an E_a_ of 33.2 kJ/mol for the esterification (Equation (4)). The experimental data aligned well with the developed reaction rate equation, considering specific conditions such as neglecting mass transfer, adsorption/desorption, and backward reaction [54]. Yang et al. examined the methyl esterification of salicylic acid (SA) using an intensified FBR. The aspects impacting kinetics are listed in Table 2 and Equation (5) [46].

(4) $$\begin{array}{l} FFA+MeOH↔FAME+H_{2}O\\ \mathrm{LHHW}\;\mathrm{model}:\\ r_{A}=-\frac{dC_{A}}{dt}=k_{1}W_{c}C_{A}C_{B},\int \frac{dX_{A}}{dt}=\int k_{1}W_{c}C_{A0}(1-X_{A})(\frac{C_{B0}}{C_{A0}}-X_{A})\\ \frac{Ln(M-X_{A})-LnM(1-X_{A})}{M-1}=k_{1}W_{c}C_{A0}t+C \end{array}$$

Kinetic equation of esterification of R. trisperma oil and methanol.

(5) $$\begin{array}{l} \mathrm{PH}\;\mathrm{model}:\;\mathrm{SA}+\mathrm{MeOH}\overset{k_{f}}{\underset{k_{r}}{⇄}}MS+H_{2}O\\ E\text{-}R\;\mathrm{model}:\;\\ r=\frac{M_{\mathrm{cat}}{(k}_{+}K_{M}a_{\mathrm{SA}}a_{\mathrm{MeOH}}{-k}_{-}K_{I}a_{\mathrm{MS}}a_{H_{2}O})}{{{(1+K}_{M}a_{\mathrm{MeOH}}{+K}_{I}a_{\mathrm{MS}})}^{2}}=\frac{M_{\mathrm{cat}}k_{f}{(a}_{\mathrm{SA}}a_{\mathrm{MeOH}}-\frac{1}{K_{e}}a_{\mathrm{MS}}a_{H_{2}O})}{{{(1+K}_{M}a_{\mathrm{MeOH}}{+K}_{I}a_{\mathrm{MS}})}^{2}}\\ \mathrm{LHHW}\;\mathrm{model}:\\ r=\frac{M_{\mathrm{cat}}{(k}_{+}K_{M}^{\prime }a_{\mathrm{SA}}a_{\mathrm{MeOH}}{-k}_{-}K_{I}^{\prime }a_{\mathrm{MS}}a_{H_{2}O})}{{\left({1+K}_{M}^{\prime }a_{\mathrm{MeOH}}{+K}_{\mathrm{SA}}a_{\mathrm{SA}}{+K}_{I}^{\prime }a_{\mathrm{MS}}{+K}_{H_{2}O}a_{H_{2}O}\right)}^{2}}=\frac{M_{\mathrm{cat}}k_{f}{(a}_{\mathrm{SA}}a_{\mathrm{MeOH}}-\frac{1}{K_{e}}a_{\mathrm{MS}}a_{H_{2}O})}{{{(1+K}_{M}^{\prime }a_{\mathrm{MeOH}}{+K}_{\mathrm{SA}}a_{\mathrm{SA}}{+K}_{I}^{\prime }a_{\mathrm{MS}}{+K}_{H_{2}O}a_{H_{2}O})}^{2}} \end{array}$$

Methyl esterification of SA with methanol is based on E-R and LHHW mechanisms.

The esterification reaction kinetics between levulinic acid (LA) and ethanol were studied in an intensified FBR using Amberlite IR120. Fluid dynamics were characterized within the reactor and the obtained kinetic data were interpreted using a dependable reactor model to account for the reaction’s extent and the fluid–solid mass transfer limitations. Kinetic models for LA esterification in continuous reactors were formulated using the gPROMS (4.0) software’s numerical method of lines. Mass balance equations were resolved via a finite difference approach, applying 200 discretization points to the packed bed’s axial coordinates [55]. A system that was independent of catalyst loading was achieved by plotting LA conversion against time and the catalyst bulk density, as outlined in Equation (6).

(6) $$\frac{dc_{LA}}{dt}=\nu_{LA}r_{LA}\rho_{B}\to -c_{LA,0}\frac{dX_{LA}}{dt}=\nu_{LA}r_{LA}\rho_{B}\to \frac{dX_{LA}}{d\left(t\rho_{B}\right)}=-\frac{\nu_{LA}r_{LA}}{c_{LA,0}}$$

The ethyl esterification of levulinic acid with ethanol.

**Figure 8:**
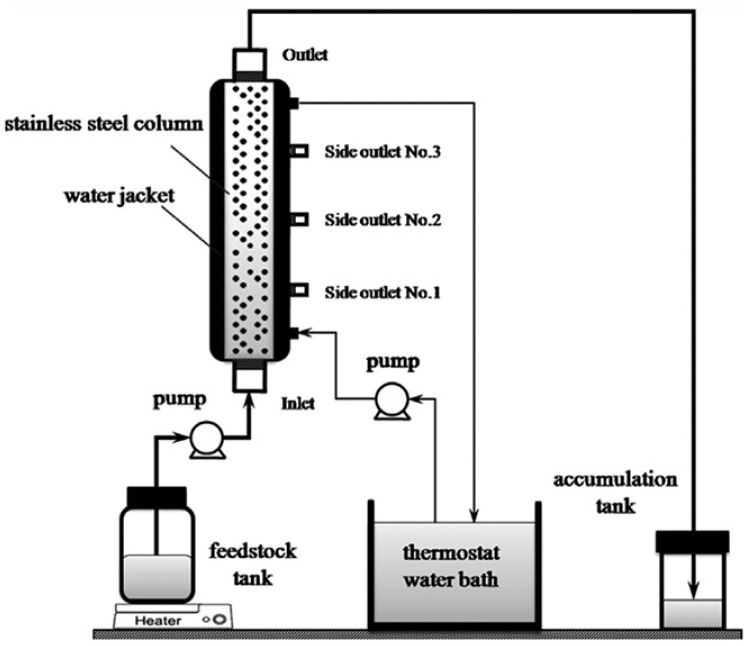
The scheme of the FBR with side outlets for continuous esterification [56].

The biodiesel production between methanol and free fatty acid (FFA) in FBRs moves from being miniaturized to pilot-scale, at 338 K (Figure 8). Under the assumption that the esterification reaction is a pseudo-homogeneous first-order reaction and ignoring the internal and external solid–liquid mass transfer resistance, the kinetics of the reaction was investigated. A kinetic model was also developed to connect FFA conversion with catalyst bed height in the FBR. Peculiarly, the model proved versatile across different resin catalysts used within the FBR, showing consistent alignment between experimental findings and theoretical predictions across all reactor scales [56].

Zhang et al. enhanced oleic acid esterification with sub/supercritical methanol using γ-Al_2_O_3_ in a PBR at optimized reaction conditions. They suggested two kinetic models: a global second-order reversible one-step model and a three-step Eley–Rideal mechanism, yielding respective E_a_ values of 83.9 kJ/mol and 9.3 kJ/mol for the reversible step (Equation (8)). The mechanism entailed three stages: oleic acid (OA) adsorption on the Lewis acid site, a methanol attack on adsorbed OA to form methyl oleate and water through esterification, and the desorption of methyl oleate from the Lewis acid site, regenerating the catalyst. Notably, γ-Al_2_O_3_ demonstrated an exceptional catalytic performance under subcritical and supercritical conditions, facilitating the maximum theoretical biodiesel yield. However, impure water hinders the esterification reaction and catalyst performance, thereby adversely affecting the conversion of biodiesel. An increased temperature and improved reactant mixing and diffusion in the supercritical state accelerate reaction rates [57]. The results of some typical studies on esterification kinetics in a micro-PBR are listed in Table 2.

(7) $$\begin{array}{l} \mathrm{OA}+*\overset{K_{1}}{⇄}{\mathrm{OA}}^{*}{,\;\mathrm{OA}}^{*}+CH_{3}OH\overset{K_{2}}{⇄}\mathrm{FAME}*+H_{2}O\\ \mathrm{FAME}*\overset{K3}{⇄}\mathrm{FAME}+*\\ r_{1}=k_{1}(C_{\mathrm{OA}}C_{*}-\frac{1}{K_{1}}C_{\mathrm{OA}*}),r_{2}=k_{2}(C_{{\mathrm{OA}}^{*}}C_{\mathrm{MeOH}}-\frac{1}{K_{2}}C_{\mathrm{FAME}*}C_{\mathrm{wat}er})\\ r_{3}=k_{3}(C_{\mathrm{FAME}*}-\frac{1}{K_{3}}C_{\mathrm{FAME}*}C_{*}) \end{array}$$

Three-step Eley–Rideal mechanism model. Lewis acid site represented as “*”.

#### 3.1.5. Catalyst-Free Esterification

**Figure 9:**
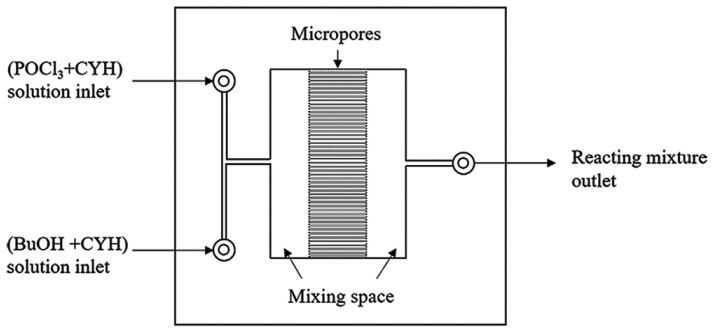
The internal structure diagram of the micromixer [58].

Monobutyl chlorophosphate (MCP), dibutyl chlorophosphate (DCP), and tri-n-butyl phosphate (TBP) can be synthesized through the utilization of POCl_3_ and n-butanol. The esterification kinetics of MCP were investigated in a microchannel system consisting of a micromixer and coiled capillaries, as depicted in Figure 9. This type of reaction kinetics follows a second-order dependence on the concentration of MCP, while displaying a first-order dependency on the concentration of n-butanol (Equation (8)). By examining the conversion rate of MCP at various temperatures, an E_a_ of 5.99 (±0.22) kJ/mol was determined, alongside a pre-exponential factor of 0.668 L^2^/mol^2^/min [58]. Notably, the reaction involved multiple elementary reactions, suggesting potential enhancements in the reaction kinetic network model for phosphate esters.


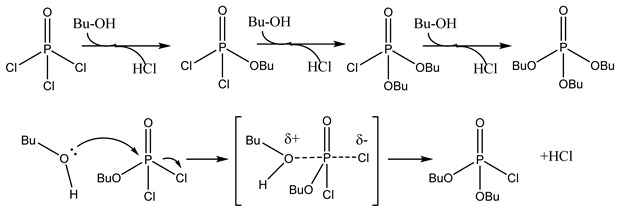

(8) $$\begin{array}{l} r=-\frac{d[MCP]}{dt}=k{\left[MCP\right]}^{\alpha }{\left[n-BuOH\right]}^{b},r=k{\left[MCP\right]}^{2}[n-BuOH],\\ \begin{array}{cc} & r=-\frac{dC_{A}}{dt} \end{array}kC_{A}^{2}C_{B},\begin{array}{cc} & C_{A} \end{array}=C_{A0}(1-X),C_{B}=C_{B0}(1-\alpha X) \end{array}$$

Kinetic equation of esterification of POCl_3_ and n-butanol, with the second-order concerning MCP and first-order concerning n-BuOH.

**Figure 10:**
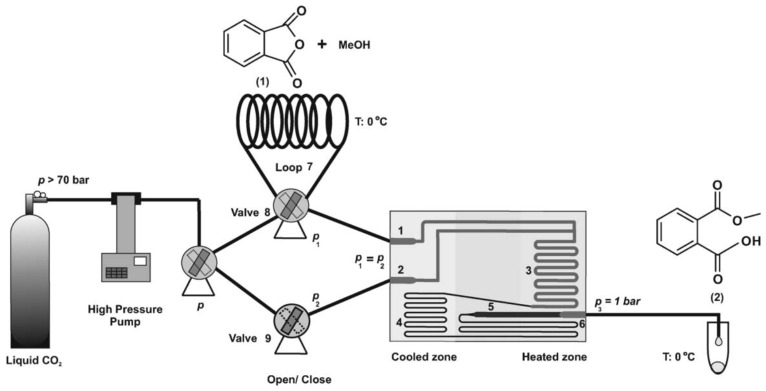
Schematic drawing of set-up for esterification reaction of phthalic anhydride with methanol at high-pressure and/or scCO_2_ conditions (90 and 110 bar). 1. Inlet reagents, 2. inlet liquid CO_2_, 3. reaction zone, 4. fluidic resistor, 5. expansion zone, 6. outlet, 7. loop that contains the reagents, 8. valve that regulates the access of the reagents to the microreactor, and 9. valve that regulates the access of CO_2_ to the microreactor [60].

C. S. Lee utilized a miniaturized intensified reactor (MIR) to evaluate the esterification kinetics of acetic anhydride and isoamyl alcohol under specified conditions. The reversible model was the most representative for this reaction. Isoamyl acetate hydrolysis had greater favorability compared to acetic anhydride hydrolysis, as indicated by a notably higher rate constant, k_3_, concerning k_4_. Water that was generated during the reaction contributed to the hydrolysis of acetic anhydride and isoamyl acetate. The reverse rate constant of isoamyl acetate, k_3_ = 0.008530 L·mol·s^−1^, significantly exceeded the hydrolysis rate constant of acetic anhydride, k_4_ = 3.983 × 10^−8^ L·mol·s^−1^, at an initial water concentration of 0.2 M [59]. Notably, despite the varying initial water concentrations, the hydrolysis rate constant for isoamyl acetate consistently surpassed that of acetic anhydride hydrolysis. Further research should consider the initial water concentration and account for non-ideal interactions to refine these findings (Equation (9)). Benito-Lopez et al. employed supercritical CO_2_ as a solvent in a continuous-flow glass microreactor to boost the reaction rate between phthalic anhydride and methanol (40 °C, 110 bar). The E_a_ was 20 kJ mol^−1^, and the A value was computed to be 410 M^−1^ s^−1^ [60]. The second-order rate constants were obtained by dividing k_obs_ by the methanol concentration at each temperature. The presence of ScCO_2_ distinctly modified the E_a_ and amplified the rate constants due to its critical conditions, which are known to promote cluster formation between reactants. This resulted in localized high-density reactant regions, forming microscopic pockets that enhanced reaction rates (Figure 10).

(9) $$\begin{array}{l} A+B\to C+D,\;C+B\to {D+H}_{2}{O,\;A+H}_{2}O\to 2C,\;A+B+C\to 2C+D\\ A=\mathrm{Acetic}\;\mathrm{Anhydride},B=\mathrm{Isoamyl}\;\mathrm{alcohol},C=\mathrm{Acetic}\;\mathrm{Acid},D=\mathrm{Isoamyl}\;\mathrm{Acetate}.\\ r_{A1}{=k}_{1}\;[A][B],r_{A2}{=k}_{2}\left[A][B][C\right],r_{r1}=-1[A][B]\\ r_{r2}{=k}_{2}{[B][C]-k}_{3}{[D][H}_{2}O],r_{3}{=k}_{4}{[A][H}_{2}O] \end{array}$$

Kinetic equation of esterification of acetic anhydride and isoamyl alcohol.

### 3.2. Kinetic of Transesterification

Transesterification involves catalyzing reactions between an ester and an alcohol/acid/ester (a different ester) and the formatting of a new ester and a new alcohol/acid/ester (Figure 2). The subsequent discussion primarily revolves around the kinetics of transesterification reactions, going beyond the realm of biodiesel production and encompassing a broader range of reactions, including those involving isocyanate and ethylene carbonate, etc. The investigation of transesterification at the micro-scale offers valuable insights into process kinetics, enabling robust predictions of process outcomes [61].

#### 3.2.1. Liquid Acid- or Base-Catalyzed Transesterification

##### The Synthesis of Biodiesel

**Figure 11:**
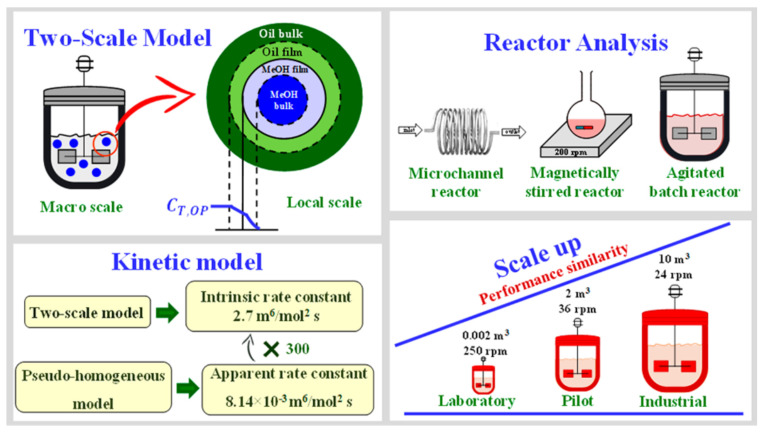
Set for the two-scale model for kinetics, design, and scale-up of biodiesel production [62].

Biodiesel is predominantly synthesized via the transesterification of renewable resources like vegetable oils or animal fats. Micro-devices, optimized to achieve the significant conversion of raw materials within shorter residence times, contrast starkly with traditional reactors. The factors influencing biodiesel synthesis—such as oil source, temperature, catalyst, raw material ratio, geometry design of microstructure reactors and reactant mixing/reaction efficiency—have been studied extensively. Diverse models have been employed to do this, including the kinetic model, reactor model, transfer models, among which the microchannel reactor model is incredibly insightful in terms of evaluating reactor performance and studying parameters like feed flow rate and residence time (as shown in Figure 11). A comprehensive two-scale model has been crafted, amalgamating macroscopic and microscopic scales to account for complex reactant, product, and catalyst interactions in biodiesel production. The validity of this model is corroborated by experimental data from microchannel and stirred-batch reactors [62].

Base-catalyzed ethanolysis of soybean oil takes place in tube reactors with T-mixer, LTF-MS, or LTF-MX. This greatly influences mass transfer and micromixing, inducing rate-constant shifts [63]. A tubular reactor packed with stainless-steel ribbon wool proves to be a promising strategy for achieving substantial soybean oil conversions to biodiesel, rivaling the efficacy of static mixers or microreactors. Strong local micromixing or narrow microchannels significantly enhance transesterification reaction rates, outstripping predictions by the pseudo-homogeneous kinetic model. The mathematical model employed hinges on the assumptions of a pseudo-homogeneous system and a uniform equilibrium constant across the reaction steps. A linear relationship exists between kinetic constants, whereby a catalyst’s CH_3_O- concentration is reduced by half in the presence of a diglyceride and by one-third for a monoglyceride. This pattern links to the extensive interfacial area development, markedly affecting methoxide anion and triglyceride molecules’ reaction and implying a liquid–liquid interface reaction. The true catalyst, comprising di- and monoglycerolates, forms in situ. Increasing the initial interface area escalates the methoxide disappearance rate, which reaches a peak when two liquid monolayers come into contact with the surface interface. The sole constraint imposed by mass transfer relates to the methanol requirements for the oil reaction phase [64,65].

Wang, X. D et al. examined FAME synthesis in a microchannel using droplet flows. The observed coalescence of neighboring droplets led to microchannel flow pattern alterations. Small-droplet coalescence into short slug droplets reduced the FAME production rate due to the decreased two-phase specific surface area. Conversely, the production rate increased when these short slug droplets fused into long slug droplets, escalating internal droplet recycling. Hence, reaching reaction equilibrium before droplet coalescence is recommended for achieving effective microreactor design and control [66].

Pontes et al. developed a mathematical model, incorporating partial differential and Navier–Stokes equations, for mass transfer within a microreactor during the transesterification of soybean oil and methanol. Their research underscores the necessity of acknowledging mass transfer restrictions in microreactor design for biodiesel synthesis. Simulation-based parametric analysis was undertaken, evaluating the effects of microreactor heights, reaction temperatures, and residence times. The goal was to decipher their influence on the transesterification process. The simulations suggested that there were enhanced triglyceride conversions at extended residence times, facilitated by the increased duration of reactant interactions. Lower microreactor heights further optimized conversions due to improved surface area-to-volume ratios. Additionally, the simulation highlighted the favorable impact of increased temperature on the process, with its effects most prominent in the microreactor’s inlet region during the early stages of the reaction [67]_._

##### The Synthesis of Ethylene Carbonate and Urethane Derivative

**Figure 12:**
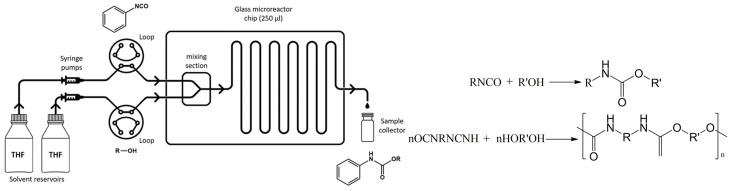
Experimental setup of microreactor system. Reaction of isocyanate and alcohol, producing urethane and polyurethane [69].

A systematic exploration of the reaction kinetic conditions that produce ethylene carbonate (EC) revealed that the mixing effect was negligible in a microreactor with a 1 mm inner diameter and flow rates between 0.60 and 0.80 mL·min^−1^. Low sodium methoxide catalyst concentrations indicated a linear relationship with the initial DMC reaction rate, whereas high concentrations showed a marginal impact. An increase in reaction temperature and an augmented EG-DMC molar ratio enhanced DMC’s consumption rate and equilibrium conversion. The endothermic reversibility of DMC’s transesterification with EG was signified by a temperature-dependent equilibrium constant, indicating enthalpy and entropy reactions (6.27 kJ·mol^−1^, 20.23 J·mol^−1^·K^−1^) [68]. Figure 12 depicts a microreactor setup used to determine kinetic parameters in phenyl isocyanate–monoalcohol reaction. Employing THF–alcohol as a solvent, the method achieved pseudo-first-order conditions due to the large excess of alcohol. The secondary alcohols’ higher energy barrier, attributed to steric hindrance from the secondary hydroxyl group, highlights reactivity differences between primary and secondary hydroxyl groups towards isocyanate. The knowledge of these kinetic parameters can guide judgements regarding when specific polymer properties should be sought [69]. Some detailed, typical studies on transesterification kinetics in microreactors are listed in Table 3.

#### 3.2.2. Biocatalytic Transesterification

**Figure 13:**
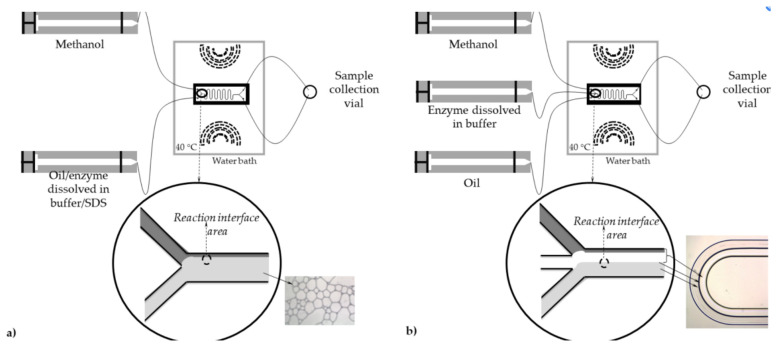
Schematic diagram of microreactor system with (**a**) two inlets and (**b**) three inlets together with picture of formed flow pattern [81].

In the study by Gojun et al., the authors compared various mathematical process models for biodiesel synthesis in a lipase-catalyzed microreactor. The 2D mathematical process model and steady-state dual parallel plug flow reactors model strongly aligned with experimental outcomes. Notably, double-substrate Michaelis–Menten kinetics were used to portray the reaction rates for transesterification and hydrolysis, with the Hill model outperforming PPBB kinetics and emerging as the superior kinetic model for biodiesel synthesis based on model selection criteria. To authenticate these models, biodiesel synthesis was performed in a microreactor under four initial conditions [80]. The substrates used included edible sunflower oil and methanol, with Lipolase L100 acting as the catalyst. Experiments were performed in microreactors with two or three inlets, as shown in Figure 13. Remarkably, when the residence time was fixed at 32 min, the FAME yield surpassed 30% in the three-inlet microreactor. The model-based mathematical simulations developed highlighted residence time as the most impactful process parameter [81]. To calculate kinetic parameters, non-linear regression was applied using experimental data and a PPBB kinetics model, yielding V_max_ = 44.71 ± 1.89 mg/mL, K_m fatty acids_ = 155.02 ± 121.86 mg/mL, and K_m methanol_ = 7.56 ± 2.77 mg/mL.

**Figure 14:**
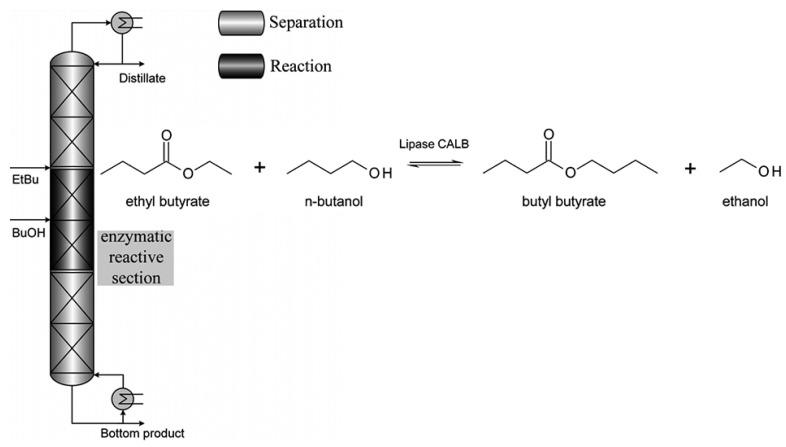
Reactive distillation column setup and transesterification of ethyl butyrate with n-butanol catalyzed by lipase CALB [82].

In another study involving the transesterification of ethyl butyrate with n-butanol in a distillation column, lipase CALB was entrapped in a hydrophobic silica xerogel matrix and then introduced as granules into the Katapak-SP-11 catalytic packing. The reaction kinetics were determined through the PPBB kinetic model and the Arrhenius model. These kinetics were used to simulate the non-equilibrium stage model of the Aspen Custom Modeler and validated on a DN50 pilot-scale column. To limit enzyme inhibition, the n-butanol concentration in the active section was minimized. Optimized conditions yielded over 90% conversion rates for n-butanol and over 26% for ethyl butyrate. The study illustrated efficient enzymatic reactions in a continuous reactive distillation column, using reduced pressure and high-temperature-tolerant lipase CALB. This is corroborated by Figure 14. The effect of these biocatalytic coatings on structured packings used for potential pilot plant application is also evaluated [82].

#### 3.2.3. Solid Acid- or Base-Catalyzed Transesterification in Micro-PBR

To acquire undistorted kinetic data from an experimental reactor, it is pivotal to curtail temperature gradients across three areas: intra-particle, interphase, and interparticle.

**Figure 15:**
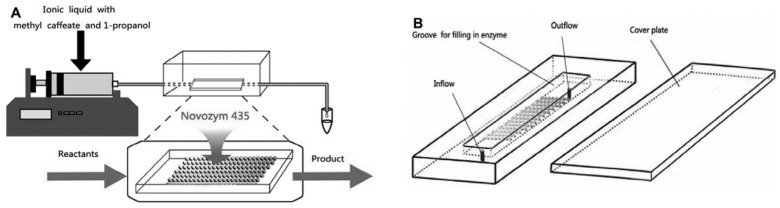
The miniaturized continuous-flow packed-bed enzyme microreactor was used to investigate propyl caffeate synthesis process parameters in the lipase-catalyzed transesterification reaction. (**A**) A diagram of the experimental setup; (**B**) the packed-bed microreactor containing Novozym 435 beads [83].

Their goal was to expedite the production of propyl caffeate from methyl caffeate and 1-propanol, using Novozym 435 in [Bmim] [CF_3_SO_3_] in PBR (Figure 15). The microreactor comprised a two-piece PDMS structure in a layered microchannel layout that was explicitly designed for transesterification. Remarkably, under optimized conditions, a peak yield of 99.5% was attained within 2.5 h, an improvement from batch reactors that demanded 24 h. The novel microreactor, with low energy expenditure and a K_m_ value 16 times lower than that of a batch reactor, proved to be an efficacious strategy for propyl caffeate production, yielding an impressive overall value of 84.0% [83]. An overview of some other studies on transesterification in micro-PBR is given in Table 3.

#### 3.2.4. Catalyst-Free Transesterification

Pontes et al. studied the kinetics and thermodynamics of the supercritical methanol transesterification of various vegetable oils into biodiesel, absent catalytic agents. The integral method was deployed to determine suitable reaction orders, adjusting experimental data to pseudo-first-order kinetic equations via the Levenberg–Marquardt algorithm. This effectively encapsulated the supercritical transesterification reaction. The calculated rate constants and Arrhenius parameters indicate the following sequence for E_a_: castor oil < jatropha oil < tobacco oil < pongamia oil < soybean oil < jojoba wax oil. This is related to the linolenic acid content; higher content leads to an increased E_a_ and reduced reaction rate [84]. Trentin et al. proposed a continuous, catalyst-free transesterification of soybean oil in a microtube reactor with sc CO_2_ as the co-solvent [70]. Catalyst-free supercritical conditions confer benefits such as enhanced production efficiency, environmental friendliness, and feedstock versatility.

For industrial applications in various esterification and biodiesel production processes, a well-planned experimental study and the subsequent development of a kinetic model are the most crucial steps. A more comprehensive and robust model can give a more significant response and provide more detail on the composition and, therefore, on the quality of the products.

A kinetic model can also be applied to obtain a better understanding of the rates of product formation and the inhibition patterns present in the transformation scheme [85]. For instance, in the enzymatic production of biodiesel, the reaction scheme can be complex, involving numerous steps and a greater number of parameters [19]. Although this complexity adds to the challenge of developing a kinetic model, once solved, such a model can be utilized to design reactors that operate based on enzyme catalysis, thereby optimizing the production process. The microreactor system for biodiesel production stands out for its efficiency, requiring a reduced workforce and lower energy inputs. This system has the potential for being scaled up to an industrial production rate of 20,589.29 L/h, delivering a high performance with a minimized risk of accident [86].

Kinetic models must be combined with reactor models. Reactor modeling is used to determine concentration, temperature, and pressure profiles, generating valuable information for the simulation and control of the scaling-up process involved.

## 4. Software Simulation

Numerical simulation is a crucial tool for analyzing the behavior of chemical species in reactors, providing quantitative and qualitative insights throughout the research and development process [3]. These simulations accurately capture mass, heat, and momentum transfer phenomena using various tailored models. While physical experimentation remains prevalent in microdevice and process optimization, integrating numerical simulation techniques, like CFD, offers a promising approach with which to enhance micro-device performance [3]. In biodiesel synthesis, computational techniques show efficacy in developing efficient micromixers that achieve optimal reagent mixing while preserving micro-scale characteristics. However, further investigations are needed to address the challenges of scaling up reaction units, with computational advancements in microdevices playing a vital role in expanding production capacities.

The Generalized Integral Transform Technique (GITT) has demonstrated efficacy in analyzing transesterification processes within microreactor systems, resolving the nonlinear mass transfer equations intertwined with chemical kinetics terms [67]. A comparison of hybrid numerical–analytical concentration fields, deduced via GITT and the results from the finite element method (FEM) through COMSOL Multiphysics (4.4) software, shows a strong correlation. Extended residence times boost triglyceride conversions due to prolonged interaction time, facilitating chemical transformations. Moreover, microreactors with diminished heights enhance triglyceride conversions, attributable to their increased surface area-to-volume ratios, while heightened temperatures are beneficial for chemical processes in the inlet region.

To calculate numerical values for the kinetic model, the least-squares method was applied within the SCIENTIST software package (MicroMath Scientist^®^ 3.0, MicroMath Scientific Software, Salt Lake City, UT, USA). Mathematica 10 (Wolfram Research) codes [81] were utilized for reactor model simulation and verification. A thorough analysis of oil, methanol, buffer phases, and their complex interplay within the microreactor system was performed using COMSOL Multiphysics (4.3b, COMSOL, Inc., Burlington, MA, USA), a finite element software.

The successful synthesis of methyl oleate in a continuous-flow rotating PBR was enabled by a custom-made nano-zinc–titanate spherical photocatalyst. The reactor’s recycling mode facilitated an autocatalytic parallel reaction mechanism, yielding an impressive methyl oleate output of 93.55%. The optimal conditions were indicated by COMSOL simulation, which exhibited excellent heat transfer and consistent temperature distribution across the reactor. Langmuir–Hinshelwood kinetics were applied to determine parameters, considering insignificant internal and external mass transfer effects. ASPEN PLUS simulation-based scale-up studies predicted a methyl oleate yield of 91.08% at a 1000-fold scale-up, closely approximating the laboratory-scale experimental yield of 93.55% [87].

**Figure 16:**
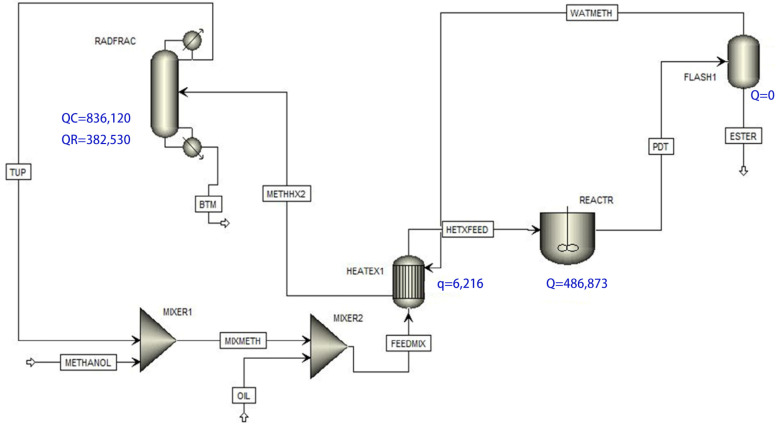
Simulated process flow diagram [88].

The synthesis of ethyl acetate catalyzed by [HSO_3_ bmim] [HSO_4_] was invested and simulated using Aspen Plus software (8.6) (Figure 16). Ideal homogeneous (IH) and non-ideal homogeneous (NIH) models were utilized, with the NIH model accurately reflecting the reaction rate, outperforming the IH model. The catalyst [HSO_3_-bmim] [HSO_4_] showed superior potential to traditional Amberlyst 15. The reaction–distillation (RD) process simulation illustrated increased ethyl acetate mass fraction with catalyst quantity, rectifying the section’s theoretical plate number and tray holdup. We ascertained the optimal ethanol feed-plate location and reflux ratio for the RD process. The reaction kinetics were effectively modeled using a pseudo-first-order forward reaction and a second-order reversible reaction [88], yielding kinetic parameters k_1_ = 0.0106 and k_2_ = 6.96 × 10^−6^ (mgKOH/g/min), with reversible reactions’ E_a_ values at 48.534 kJ/mol and 18.744 kJ/mol, respectively. The simulation indicated a notable 99.85% oleic acid conversion under analogous experimental conditions.

## 5. Microreactions Online

Microreactors serve as innovative production units as well as efficient laboratory tools for comprehensive chemical process kinetic studies with online analyses. The fusion of microreactors and spectroscopic analyses is an emerging, fast-evolving field, and numerous studies have been conducted exploring this interface.

Microreactors are lauded for their accurate control over process conditions. With distinct flow patterns, exceptional mixing, even temperature distribution, and broad, safe operational parameters, they offer a unique platform for kinetic data extraction. Numerous techniques have been devised for integrating spectroscopic detection into these continuous processes, permitting real-time reaction monitoring. This feature serves a dual purpose—optimizing reaction parameters and deriving vital kinetic data.

Online reaction monitoring allows for rapid data acquisition, thereby exposing the existence of short-lived species or unstable intermediates that may evade detection through conventional offline analysis [25].

McMullen et al. integrated Attenuated Total Reflectance FTIR (ATR-FTIR) spectroscopy, Raman spectroscopic, fluorescence correlation spectroscopy, small-angle X-ray scattering (SAXS), and nuclear magnetic resonance (NMR) spectroscopy with microreactors for process analysis and kinetic study [59,89,90,91,92]. NMR offered insights into the kinetics of esterification reactions by providing well-defined reactor models of the probe head [93]. Online spectroscopic detection demonstrated immense potential for microreactor process optimization. A prime example is the acid-catalyzed esterification of ethanol and acetic anhydride to ethyl acetate within a polydimethylsiloxane (PDMS) microreactor chip [25]. This process combines confocal laser scanning microscopy using laser-induced fluorescence (CLSM-LIF) with computational fluid dynamics (CFD) simulation, which offers reliable concentration distribution insights within a micro-mixing device, specifically at low Reynolds numbers (30 < Re < 120). In this context, radial secondary structures like Dean vortices enhance diffusive mixing, reducing mixing length. It was established that the most efficient mixing occurs with an inlet geometry along the outer channel wall. CLSM-LIF integration helps to understand the complex interplay between flow pattern, flow rate, reactor geometry, product yield, and selectivity. The CFD model aids in identifying areas of incomplete mixing, where fluid viscosity rises due to competitive-consecutive esterification of di-isocyanate reaction, potentially causing fouling [94,95].

The amalgamation of real-time Fourier transforms infrared (FTIR) analysis with multi-step diazo group transfer, extraction, separation, and diazo decomposition, as well as X-H insertion reactions, significantly streamlines kinetic parameters determination. This is essential for formulating reactors and mechanistic models [96]. By developing an optimized liquid/liquid flow separation method, the necessity for liquid chromatography is bypassed, yielding highly pure aryl diazo acetates. The E_a_ and reaction order data about the reactant species or solid acid catalysts coincide with the previously documented literature [97].

The benefits of faster data collection include the deeper monitoring of chemical transformations, the quicker optimization of reaction conditions, and a reduction in the time and waste associated with optimizing synthetic transformations. The generation of large volumes of data at a fast pace paves the way applying advanced statistical techniques for process analysis, such as ‘the Design of Experiments’ (DoE), allowing for simultaneous scrutiny of multiple parameters.

Chief among these is process control, using data generated during the transformation to modify key input parameters such as residence times, temperatures, and pressure, optimizing output parameters including yield, selectivity, and productivity.

The next frontier involves the incorporation of feedback optimization algorithms. These algorithms can autonomously interpret real-time data, identifying regions within the parameter space that either maximize or minimize outputs of interest. Utilizing these advanced systems allows the experimental loop required for optimizing processes to be closed with minimal human intervention, accounting for unforeseen changes in the inputs, possibly arising from physico-chemical variability in the feed stocks.

The amalgamation of automated flow chemistry, real-time analytics, and optimization algorithms offers substantial potential, potentially enabling the synthesis of virtually any molecule with limited human intervention [13]. These experiments rely on automated systems, chemo-informatics software, and decision-making algorithms. Claudia et al. delve into robotic experiments, 3D-printed labware, multi-instrument systems, and machine learning optimization algorithms [98,99].

Microreactor technology demonstrates potential in biological research, notably in enzyme kinetics and inhibition studies using acetylcholinesterase (AChE) and angiotensin-converting enzyme (ACE) model systems. This is achieved by integrating a microreactor with continuous spectrophotometric detection in Sequential Injection Lab-on Valve (SI-LOV) mode. The method provides real-time kinetic measurement, automated mixing, initial reaction rate monitoring, and kinetic constant determination for enzymes and inhibitors, expanding microreactor technology’s applications across scientific fields [92].

**Figure 17:**
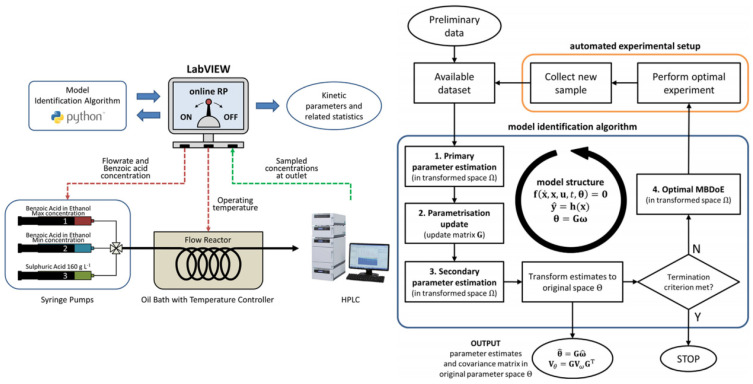
Simplified diagram representing the online model identification platform. Proposed framework for the online identification of models in automated model identification platforms [100].

Federico Galvanin’s key contribution is an online model reparametrization (RP) strategy, a tool that automates model parameter space transformation during online model identification, enhancing robustness against numerical failures caused by model sloppiness in unmanned platforms. Assuming the user provides appropriate kinetic model equations at the campaign’s onset, the advantage of online RP is demonstrated in an experimental study, aiming to identify the kinetic model of benzoic acid’s catalytic esterification in a microreactor system [100] (Figure 17). The fundamental step in the procedure is updating the parametrization matrix G after collecting and fitting each sample. The online modification of the model parametrization is performed to maintain a high computational performance at the parameter estimation and optimal MBDoE stages in the procedure.

## 6. Automation and Digitization Reaction

The autonomous platform was developed using a combination of LabVIEW and Python, where LabVIEW was used to control lab equipment and monitor process values and Python was used to perform online parameter estimation, online MBDoE, and also to transcribe the experimental results to Excel files. It also demonstrated its effectiveness across three distinct scenarios: conducting user-defined steady-state experiments, employing real-time optimization of conditions using MBDoE algorithms for enhanced parameter precision, and performing transient experiments under user-selected conditions. Notably, experiments performed using online MBDoE algorithms offer more accurate parameter estimates than traditional factorial design, demonstrating the value of online parameter estimation and MBDoE in steady-state experiments [101].

Transient experiments, on the other hand, offer advantages like accelerated parameter estimation and reduced reagent usage by avoiding steady-state conditions. Despite lower precision in parameter estimates compared to steady-state experiments, transient experiments notably diminish the overall experiment duration, being completed in just two hours instead of a steady-state campaign’s eight hours [12].

The integration of flow and analytical platforms provides one notable advantage: the ability to enhance process control by utilizing the data generated throughout the complex chemical transformations. These data can be employed to modify input parameters, such as residence times, temperatures, and pressure, to optimize the desired output of the transformation, which may include factors like yield, selectivity, and productivity. Taking a step further, the evolution of these platforms involves integrating feedback optimization algorithms that autonomously interpret real-time data. These algorithms can effectively identify regions within the parameter space that maximize or minimize the desired outputs of interest. To achieve this, various self-optimization algorithms are employed, including the stable noisy optimization by branch and fit (SNOBFIT), the simplex and steepest descent, the super modified simplex algorithm (SMSIM), and the multi-objective active learner (MOAL) [102].

The conjunction of automated continuous-flow systems with online analysis and feedback presents possibilities in modeling and optimizing chemical syntheses with scarce pre-existing reaction data. This advancement optimizes product yield or selectivity within multi-step reaction networks typically characterized by low desired product formation selectivity [103].

One significant achievement involves developing an automated system capable of rapidly estimating accurate kinetic parameters using maximum likelihood estimation (MLE) and a D-optimal experimental design, demonstrated with the Langmuir–Hinshelwood model. Online MBDoE reduces the number of experiments needed for model discrimination and precise parameter estimation. A single MBDoE-designed experiment can distinguish between two models, enhancing parameter estimate accuracy and minimizing prediction uncertainty. The autonomous, closed-loop system enables all kinetic experiments to be completed in just three days, with parameters estimated via MLE, taking into account the negligible reverse reaction due to a large ethanol surplus, showcasing the platform’s flexibility and adaptability [9]. 

In the scientific realm, automated flow systems’ continuous evolution and development necessitate further exploration. The focus should be on acquiring additional knowledge within the reaction system and designing intelligent systems capable of diagnosing, modeling, and parameterizing reaction networks without relying on user-supplied models. Rapid online measurement tools like flow infrared spectroscopy (Flow IR) are invaluable for gaining extensive knowledge within the reaction system, enabling the diagnosis of by-product formation dynamics. While individual capabilities for diagnosing, modeling, and parameterizing reaction networks have been demonstrated, constructing an automated system capable of proposing novel mechanisms from experimental data and validating them through tests remains a challenge. To address this challenge, designing a robust a priori system capable of experimenting under conditions characterized by high parameter sensitivity is crucial. This often involves isolating reactions and intermediates and operating under challenging reaction conditions [5]. 

The fusion of automated systems with online feedback in continuous-flow technology provides a promising avenue for parameterizing and optimizing chemical syntheses, even with sparse prior reaction data. This approach mitigates reagent usage and delivers vital data for reaction scale-up. For exceptionally intricate chemistry, however, a demonstration of resource-efficient, effective platforms, and procedures that can parameterize complex reaction networks is crucial. Robotic microreactors, supplemented by real-time data-driven feedback loops, provide the perfect setting for online modeling and optimization, blending automation, and digitization. Optimal experimental design contributes significantly by allowing the strategic planning of future process conditions based on current data and objectives. In the presence of multiple conflicting objectives, an equitable compromise may be the most effective solution [104,105].

**Figure 18:**
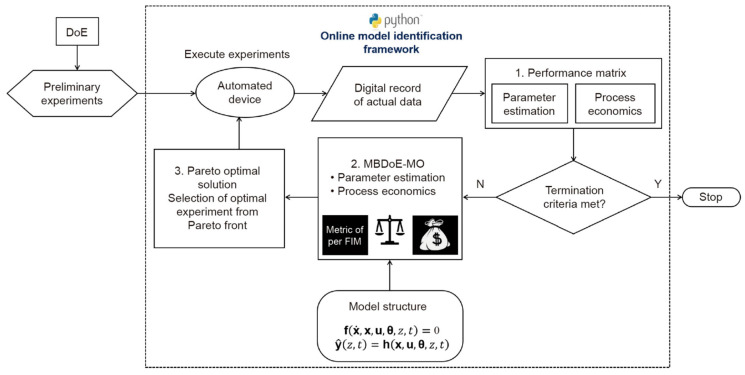
Proposed framework for online multi-objective optimal experimental design in automated model-identification platforms. Framework is used to design experiments that improve precision of online parameter estimation with minimum experimental cost [32].

This study presents an online multi-objective optimal experimental design framework to identify beneficial trade-off solutions within multiple constraints. Employing a decision-making step and a Fisher Information Matrix (FIM)-based metric, the framework selects the optimal Pareto point for subsequent experiments. The merits of this framework are demonstrated in a simulated case study of identifying a kinetic model for benzoic acid esterification. The results underscore the superiority of the Model-Based Design of Experiments Multi-Objective (MBDoEMO) approach in reaction kinetic studies in steady-state flow systems (Figure 18), enabling the recognition of trade-offs that enhance information extraction while minimizing material usage. As a general Python function, this framework can extend to various real online multi-objective optimization problems [32].

An autonomous reactor platform expedited the identification of a kinetic model for Amberlyst-15 catalyzed esterification of benzoic acid with ethanol (Equation (10)). Exhibiting plug flow and efficient mass transfer, the platform streamlined catalytic kinetic studies, reducing experiments required for kinetic model identification [9]. The methodology encompassed: (i) factorial design for potential kinetic model identification; (ii) model proposal and data-based examination; (iii) identifiability analysis to refine estimable models; (iv) online MBDoE for model selection; and (v) parameter precision enhancement via MBDoE.

(10) $$\begin{array}{l} r_{\mathrm{BA}}^{\prime }{=-\mathrm{kC}}_{\mathrm{BA}}C_{\mathrm{EtOH}},r_{\mathrm{BA}}^{\prime }=\frac{-kC_{\mathrm{BA}}C_{\mathrm{EtOH}}}{{\left(1+K_{W}C_{W}\right)}^{2}},\\ r_{\mathrm{BA}}^{\prime }=\frac{-kC_{\mathrm{BA}}C_{\mathrm{EtOH}}}{{\left(1+K_{W}C_{W}+K_{\mathrm{EtOH}}C_{\mathrm{EtOH}}\right)}^{2}},r_{\mathrm{BA}}^{\prime }=\frac{-kC_{\mathrm{BA}}C_{\mathrm{EtOH}}}{{\left(1+K_{BA}C_{BA}+K_{\mathrm{EtOH}}C_{\mathrm{EtOH}}+K_{W}C_{W}+K_{EB}C_{EB}\right)}^{2}}\\ k=\exp(-kP_{1}-\frac{KP_{2}\times 10,000}{R}\times [\frac{1}{T}-\frac{1}{T_{M}}])\\ A=\exp(-kP_{1}+\frac{KP_{2}\times 10,000}{RT_{M}}),Ea=KP_{2}\times 10,000\\ {r^{\prime }}_{BA}=\frac{185.3\exp(-\frac{68,800}{RT})\times C_{BA}C_{EtOH}}{{\left(1+0.53C_{W}\right)}^{2}} \end{array}$$

Kinetics of alkali catalyzed esterification of benzoic acid with ethanol using autonomous microreactor platform.

**Figure 19:**
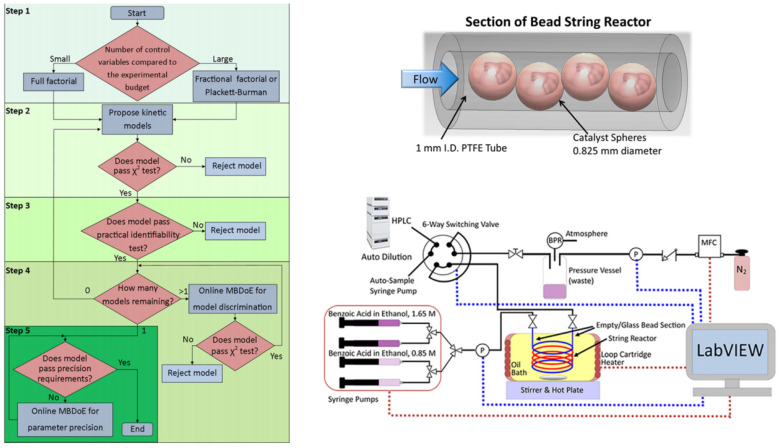
Flowchart of the general methodology proposed for kinetic model identification. Section of the bead string reactor showing the PTFE tubing with 1.58 mm outer diameter and 1 mm inner diameter, filled with 0.825 mm diameter Amberlyst-15 spheres. Autonomous reactor platform used for the kinetic experiments for the esterification of benzoic acid with ethanol usingAmberlyst-15 as a heterogeneous catalyst in a string reactor. Red dashed lines indicate that LabVIEW controls the equipment, and blue dashed lines indicate that LabVIEW reads the measurement from the equipment. BPR, MFC, and P indicate the back-pressure regulator, mass flow controller, and pressure sensor, respectively [9].

Kinetic experiments were executed using a closed-loop autonomous reactor platform (Figure 19). The final rate expression for the esterification reaction is given in the above equation, with the determined values for the A, apparent E_a_, of water as 185.3 L^2^/(g·s·mol), 68.8 kJ/mol, respectively [9].

## 7. Conclusions

This literature review summarizes the progress made in esterification and transesterification kinetics in microreactors. Efficient heat transfer and precise time control facilitate kinetic measurements in fast reactions with transition intermediates. Heat-mass transfer plays a pivotal role in esterification, impacting yield and selectivity. Microfluidic technology minimizes reactant consumption and eliminates dead volume, enhancing the process. In microstructured reactors, the determination of intrinsic kinetics for esterification with heterogeneous catalysis becomes more feasible due to intensified heat-mass transfer and increased surface-to-volume ratio, especially in fixed-bed configurations. Mass transfer limitations govern transesterification, be it base- or acid-catalyzed. Improved micromixing efficiency, reduced microreactor diameters, and higher flow rates enhance mass transfer. The reactor type significantly affects mass transfer, inducing shifts in Ea and rate constants through micromixing. In biocatalysis of esterification and transesterification, immobilization support can be achieved via microreactors or solid carriers, effectively enhancing enzyme stability and productivity.

Automated continuous-flow systems, and online analysis, enhance chemical synthesis modeling and optimization, even with limited reaction information. These systems expedite kinetic experiments, enabling model identification with fewer trials, thus significantly increasing productivity. It can also be used to find optimal step-sizes for gradient-based optimization techniques.

Simplifications can result in inaccurate kinetics, low reproducibility, and limited reactivity comprehension. Factors like supercritical conditions, charged species, and counterion interactions, varying concentrations of off-cycle catalytic species, and competitive reactions all contribute to these complexities.

Looking ahead, the integration of artificial intelligence into micro-platforms to achieve human-machine interaction dialogue, online data collection, closed-loop feedback algorithm, and self-optimizing experimental conditions consistency. It holds great promise for addressing various problems in continuous microreaction kinetics processes. This can enhance our ability to overcome the limitations mentioned above and enable more efficient and accurate modeling and optimization of continuous-flow systems. Integrating artificial intelligence into micro-platforms, the entire process can be achieved in a closed and controllable manner. This technology has the potential to be prioritized for non-gravity outer space chemical material experiments.

## Figures and Tables

**Table 1:** Michaelis–Menten, Lineweaver–Burk and Lilly–Hornby kinetic models.

Ref	Model	Equation	Description
[20,21]	Michaelis– Menten	$V_{0}=\frac{V_{\max}[S]}{\left(K_{m}+[S]\right)}=\frac{K_{cat}[E][S]}{\left(K_{m}+[S]\right)}$ $V_{0}=V_{\max}=k_{cat}[E]$	V_0_, reaction rate; V_max_, the maximum rate; [S], substrate concentration; [E], enzyme concentration; K_cat_, the turnover number; K_m_, Michaelis–Menten constant; Q, flow rate; C, reaction capacity of the continuous-flow packed-bed microreactor; [A], [B] is the substrate A, B concentration; k_1_, the rate constant; f, the fraction of the substrate converted to the final product during the reaction.
[22]	Lineweaver–Burk	$\frac{1}{V_{0}}=\frac{K_{m}}{V_{\max}}\cdot \frac{1}{\left[S\right]}+\frac{1}{V_{\max}}$	
[23]	Lilly–Hornby	$f\cdot [A_{0}]=\frac{C}{Q}+K_{m(app)}\cdot Ln(1-f)$	
[24]	Ping Pong Bi Bi	$v=(k_{1}*[E]*[A])/(K_{mA}*(1+[B]/K_{mB}))+[A]$	

**Table 2:** An overview of some typical studies on esterification kinetics in microreactors.

Entry	Reactants	Catalyst	Kinetic Equation	Microreactor	E_a_/kJmol^−1^
[31]	Propionic acid, 1-butanol	Lipase	$v_{0}=\frac{V_{\max}}{1+(K_{A}/[A])(1+([B]/K_{B}))+(K_{B}/[B])}$	Microchips	22
[32]	Benzoic acid, ethanol	Sulfuric acid	$\begin{array}{l} v\frac{dC_{i}}{dz}=v_{i}kC_{BA},\forall i=1,\ldots ,N_{y}\\ k=\exp[\ln A-\frac{(E_{a}/10^{4})10^{4}}{RT}] \end{array}$	Microreactor platforms	78.5
[33]	Benzoic acid, ethanol	Sulfuric acid	r_BA_ = −kC_BA_ r_BA_ = −kC_BA_^2^	PEEK tube	79.8
[34]	4-ethyl-neoxetan-2-one, methanol	TsOH	$(-r_{A})=-\frac{dC_{A}}{dt}=KC_{A}^{\alpha }C_{B}^{\beta }=KC_{A}^{\alpha }{\left(C_{A}+0.3C_{0}\right)}^{\beta }$	Helical microreactor	-
[35]	Ethanol, hexanoic Acid	H^+^ membrane	r = kC_HA_C_Ethanol_	Membrane Reactor	-
[36]	Ethylene glycol and acetic acid	Amberlyst 15	r = kC_EG_C_AC_	Tubular reactor	53.21
[37]	sodium 4-tbutylphenolate, 4-methoxybenzoyl chloride	PTC (1 M NaOH, CH_2_Cl_2_)	$-\frac{dc_{RX}(t)}{dt}=k_{org}c_{RX}^{a}(t)c_{QY}^{b}(t)$	PEEK microtube	-
[9]	Benzoic acid, ethanol	Sulfuric acid	$r_{BA}^{\prime }=\frac{185.3\exp(-\frac{68.800}{RT})\times C_{BA}C_{EtOH}}{{\left(1+0.53C_{W}\right)}^{2}}$	PBR	79.9
[38]	Acetic acid, Isoamyl alcohol	NKC-9	$\begin{array}{l} r_{PH}=M_{cat}(k_{f}a_{HAC}a_{IAA}-k_{r}a_{IAA}C^{a}H_{2}O)\\ =M_{cat}k_{f}(a_{HAC}a_{IAA}-\frac{1}{K_{e}}a_{IAA}C^{a}H_{2}O) \end{array}$	PBR	PH model, 58.6, 65.3
[39]	Acetic acid, isopropyl alcohol	Amberlyst 36 Wet	$\begin{array}{l} r_{PH}=M_{cat}(k_{f}a_{HAC}a_{IPA}-k_{r}a_{IPA}a_{H_{2}O})\\ =M_{cat}k_{f}(a_{HAC}a_{IPA}-\frac{1}{K_{a}}a_{IPA}a_{H_{2}O}) \end{array}$	PBR	LHHW model, 64.56, 66.94
[40]	Acrylic acid Ethanol	Amberlyst 35	IQH, NIQH, ER, LHHW, modified ER, modified LHHW models	PBR	78.72, 73.23 59.26, 62.34 53.89, 59.6
[41]	Geraniol, propionic acid	Novozym 435	$v_{0}=\frac{V_{\max}[A][B]}{K_{mB}[A]+K_{mA}[B](1+\frac{\left[B\right]}{K_{iB}})+[A][B]}$	PBR	2.4 kcal/mol
[42]	Nonanoic acid esterification 2-ethylhexanol	Amberlyst-15, IR120	$\frac{\partial c_{i}}{\partial t}=-u\frac{\partial c_{i}}{\partial z}+Dz\frac{\partial^{2}c_{i}}{\partial z^{2}}-k_{s}a_{sp}(c_{i}-m_{i}c_{is})$	PBR	91.2, 57.7
[43]	Methacrylic acid, methanol	NKC-9 resin	$-r_{A}=\frac{k_{r}(x_{A}x_{B}-x_{C}x_{D}/K_{eq})}{{\left(1+K_{A}x_{A}+K_{B}x_{B}+K_{C}x_{C}+K_{D}x_{D}\right)}^{n}}$	PBR	P-H 50.25 E-R 53.42 L-H 56.71
[44]	n-Butanol, levulinic acid	lipases	Lilly-Hornby model $f\cdot [A_{0}]=\frac{C}{Q}+K_{m(app)}\cdot \ln(1-f)$	PBR	7.07 kcal/mol
[45]	Oleic acid, methanol	alkaline	$r=K_{C}x_{A}x_{M}\{1-(1/K_{e})[(x_{E}x_{W})/(x_{A}x_{M})\}C_{cat}$	PBR	14 kcal/mol
[46]	Salicylic acid methanol	NKC-9	PH, ER, LHWW	PBR	39.24, 41.22; 44.83, 46.81
[47]	2-ethylhexanoic acid, ethanol	None	Unsteady-state diffusion model	microchannel	-

**Table 3:** An overview of typical studies on transesterification kinetics in microreactors.

Entry	Reactants	Catalyst	Kinetic Equation	Microreactor	E_a_/(kJ/mol)
[70]	Soybean oil, ethanol	Catalyst-free	$\frac{dTAG}{dt}=-k_{1}\cdot TAG\cdot EtOH+K_{2}\cdot DAG\cdot FAEE$	Microtube reactor	-
[63]	Soybean oil, ethanol	KOH	$\begin{array}{l} r_{1}=k_{1}{\left[T\right]}^{P}[EtOH][EtO^{-}]\\ r_{2}=k_{2}\times {\left[SB\right]}^{P}[EtOH][EtO^{-}] \end{array}$	Mixer, PTFE tube	-
[71]	Methanol, soybean oil	NaOH	$R_{TG}=-k_{1}C_{TG}C_{MeOH}+k_{2}C_{DG}C_{M}$	Single-channel microreactor	-
[72]	Diethyl oxalate methanol	K_2_CO_3_	$-r=\frac{dC_{DEO}}{dt}=kC_{DEO}^{\alpha }C_{MeOH}^{\beta }$	Micromixer, tail tube	31.86
[73]	Vegetable oil, ethanol	EtONa	$\begin{array}{l} \frac{d[EE]}{dt}=k_{1}{\left[TG\right]}_{E}[EtOH][Cata]-k_{-1}{\left[DG\right]}_{E}[EE][Cata]\\ +k_{2}{\left[DG\right]}_{E}[EtOH][Cata]-k_{-2}{\left[MG\right]}_{E}[EE][Cata]\\ -k_{3}{\left[MG\right]}_{E}[EtOH][Cata]-k_{-3}[G][EE][Cata] \end{array}$	PFA tubes	-
[74]	(R,S)-2-pentanol, acyl donor	Novozyme 435	$r^{\prime \prime }=\frac{4.16C_{R}C_{A}}{51.17C_{R}+103.73C_{A}+C_{R}C_{A}}$	continuous- flow reactor	-
[75]	Soybean oil, ethanol	Supercritical conditions	$v_{\mathrm{int}}=v_{\sup}=v_{\max}[1-{\left(\frac{r}{R}\right)}^{2}]$	Tubular reactor	32.8 39.98
[76]	Propylene glycol, methyl ether, ethyl acetate	Amberlitetm IRA904	$\frac{dc_{A}}{dV_{r}}=\frac{-\eta (1-\varepsilon )\varepsilon_{\mathrm{CALB},P}({\hat{k}}_{1}[c_{As}^{2}(1-K)+c_{A_{s}}(c_{B_{0}}-c_{A_{0}}+2Kc_{A_{0}})-K(cA_{0}^{2}])}{F_{v}}$	Chromatogra- phic reactors	-
[77]	Waste cooking oil, methanol	Calcium oxide	$-\ln(1-X_{ME})=kt$	PBR	45.72
[78]	Oleic acid, ethanol	Niobium phosphate	$y=-9.79w_{1}^{2}-4.87w_{2}^{2}-0.93w_{3}^{2}+63.73$	PBR	-
[79]	Malonic acid, citronellol	Amberlite MB-1	$\frac{r}{r_{\max}}=\frac{\left[A\right]}{\alpha K_{A}(1+(K_{B}/[B])+(K_{B}/Ki)+([B]/\beta K_{i}))+[A](1+\alpha K_{B}/[B]))}$ Lilly-Hornby	PBR	-
[23]	Ethyl ferulate glycerol	CALB lipase		3D-printed polylactic acid	K_m(app)_ 3.68 mM
[55]	Palm oil methanol	NaOH	${-R}_{\mathrm{TG}}{=w}_{cat}(k^{+}C_{TG}C_{M}-k^{-}C_{GL}C_{ME})$	Microchannel	76.97 106.23
[59]	DMC ethylene glycol	Sodium methoxide	$\frac{1}{v_{i}}\frac{dC_{i}}{dt}=C_{cat}(K_{+}\alpha_{DMC}\alpha_{EG})-\frac{K_{+}}{K_{eq}}\alpha_{EC}\alpha_{MeOH}^{2}$	Tube reactor	4.294 × 10^4^ 3.668 × 10^4^
[69]	PhNCO, alcohol			Microchannel	30.4, 38.1, 30.2, 38.6

## Data Availability

The data presented in this study are available on request from the corresponding author due to privacy.

## Abbreviations

AC	Acetic acid	MAP	maximum a posteriori
Ea	activation energy	MNPs	magnetic nanoparticles
BOH	n-butanol	MIR	miniaturized intensified reactor
C_i,_ i = A, B, C…	Concentration of Component i	MCP	monobutyl chlorophosphate
CAPE	caffeic acid phenethyl ester	PPBB	Ping Pong Bi Bi
CA	caffeic acid	PBR	packed-bed reactor
CALB	Candida Antarctica lipase B	PES	polyethersulfone
CFD	computational fluid dynamics	PE	2-phenylethanol
DCP	dibutyl chlorophosphate	PEEK	polyether ether ketone
DoE	Design of Experiments	P	Pressure
DMC	dimethyl carbonate	PTFE	polytetrafluoroethylene
EG	ethylene glycol	PerFluoroAlkoxy	PFA
EC	ethylene carbonate	PTSA	p-toluene sulfonic acid monohydrate
FAME	fatty acids methyl esters	PTC	phase transfer catalyst
FIM	Fischer information matrix	RD	reactive distillation
FBR	Fixed-bed reactor	RTD	residence time distribution
FFA	free fatty acid	SciPy	the scientific Python
GC	gas chromatography	SNOBFIT	the stable noisy optimization by branch and fit
GITT	Generalized Integral Transform Technique	SMSIM	the super modified simplex algorithm
HA	Hexanoic Acid	A	pre-exponential factor
Lipolase L100	thermomyces lanuginosus	SAXS	small-angle X-ray scattering
LA	levulinic acid	SQP	sequential quadratic programming
MBDoE	Model-Based Design of Experiments	SA	salicylic acid
MBDoE-PE	MBDoE methods for improving parameter estimation	sc CO_2_	Supercritical CO_2_
MBDoE-MO	multi-objective MBDoE	T	Temperature
MOAL	the multi objective active learner	TBP	tri-n-butyl phosphate
MLE	maximum likelihood estimation	TsOH	4-methylbenzenesulfonic acid
